# Progress in Gynæcology

**Published:** 1897-04-24

**Authors:** 


### Progress in Gynæcology.

(*Continued from page* 48.)

**Puerperal Eclampsia.**—Attention was drawn to the necessity of proving whether the child was alive or not before deciding on interference by J. W. Byers,[7] since both albuminuria and convulsions are put an end to by the death of the fœtus, while, on the other hand, the tendency to eclampsia is greater in multiple pregnancies, the maternal mortality being highest when the convulsions come on during pregnancy, less when they occur during labour, and least of all when they occur after labour, hence is argued the importance of the waste products produced by fœtal growth. The views to account for the occurrence of eclampsia may be grouped under three heads—(1) The reflex nervous theory from irritation, due to enlargement and contraction and retraction of the uterus; this theory is untenable. (2) The theory that the convulsions are caused by kidney disease. The condition of the urine indicates that there is interference with the renal function, and often disease of the kidneys is found; but, in some cases, gross lesions of the kidneys are absent, and albuminuria also. Moreover, eclampsia does not always occur in cases of chronic renal disease, and the certainty of the occurrence of eclampsia is not proportionate to the albuminuria. Convulsions, on the other hand, are rare in acute inflammations of the kidneys, apart from pregnancy, except when these latter occur in the course of certain specific fevers. Albuminuria is a predisposing, but not the exciting or sole cause, of eclampsia. (3) The toxæmic hypothesis. This is favoured because it covers all cases, whether eclampsia is present or no. The clinical history of eclampsia also resembles that of a toxæmia, while the post-mortem appearances point to the existence in the circulation of some blood-coagulating product of organic change, either creatin, some leucomaine, or carbonic acid; thrombosis being invariably found in the liver, lungs, and brain in all fatal cases.

This theory is of great assistance in the prophylactic treatment of eclampsia. Tarnier states that the improvement in the results of cases treated by him is due to the adoption of this hypothesis, and the basing of his treatment—milk diet and stimulation of elimination—on its logical indications.

Van de Velde[8] endeavours to give the last theory an experimental basis. He compared the ease with which human urine produced convulsions in pregnant and non-pregnant rabbits. In the pregnant 9 c.cm., in the non-pregnant 20 c.cm. per kilogr. of body-weight, on the average, induced convulsions. He only failed once in 37 cases in inducing convulsions. If the blood of a pregnant and non-pregnant animal be injected at different times into the same rabbit, 18 c.cm. of that of the pregnant animal will produce convulsions, as against 25 c.cm. (per kilogram of body weight in both cases) of that of the non-pregnant. If urine be substituted for blood the quantities are 18 and 30 c.cm. per kilogr. respectively. This appears to be strong evidence in favour of the auto-intoxicative toxæmic theory.

The necessity of the routine examination of the urine of all women in the last two months of pregnancy, showing any trace of œdema, especially of primiparæ, and early prophylactic treatment is insisted on by Charles,[9] while the rarity of eclampsia in cases where sickness continued throughout pregnancy is noted by W. S. Philips.[10] This points to the influence of ingestion on the accumulation of waste products in the blood, especially when the eliminatory processes are lowered. He also notes the low specific gravity of the urine in many cases. The frequency of eclampsia in primiparæ may be accounted for by the adoption of better prophylactic treatment in the subsequent pregnancies of those who have once suffered.

The danger does not depend on the amount of albumen present, but more on the amount of solid waste products excreted daily (especially urea). This cannot be calculated from specific gravity alone, but from a careful estimation of total weight of waste products present per diem. That arterial pressure is an important indication is shown by Vaquez and Nobécourt.[11] It remains normal in the pregnant until labour commences, but is as high as 18-20 cm. in albuminuric pregnant women; it rises with the attacks and falls after them, the attack is preceded by a rise, and rises to an average higher level with repeated attacks.

The treatment recommended has the following objects: (1) The diminishing of waste products of ingestion, and so relieving the strain on the eliminatory organs; and (2) the increase of elimination by stimulation of those which can act as accessories to the kidney. The first result is achieved by the strict dieting of a suspected eclamptic patient, *i.e.*, a pregnant woman suffering from œdema or albuminuria, the diet recommended by Charpentier, Tarnier, and others being entirely confined to milk; this has given excellent results in the hands of the writers mentioned. Under the second heading come the treatment by diuretics, diaphoretics, and purgatives; these call for no remark, except to mention that pilocarpin is most dangerous as a diaphoretic in this condition, on account of the increased bronchial secretion and bronchial œdema produced by it. The diminution of the explosive attacks by sedatives, as chloral, morphia, and chloroform, is another important object, while if cyanosis with pulmonary engorgement is a symptom, free blood-letting is of the greatest benefit.

Whether or no pregnancy should be at once terminated is a much debated point. Tarnier and the French school advise dealing with the convulsions by blood-letting, chloral, the inhalation of chloroform, and subcutaneous or intravenous injection of normal saline solution; the German school, Halbertsma, Mangiagalli, and others, advocate the early emptying of the uterus, this is supported by Edgar.[12] Dührssen recommends division of the os, if necessary, to expedite delivery.

[7] Lancet, Jan. 2, 1897. [8] B. M. J. Epit., Feb. 6, 1897. [9] B. M. J. Epit., Nov. 21, 1896. [10] Columbus Med. Journ., Jan. 19, 1896. [11] Medical Week, Feb. 5, 1897. [12] New York Med. Record, Dec. 26, 1893.

